# Diagnostic Performance of Contrast-Enhanced Computed Tomography in Predicting Lymph Node Metastasis in Epithelial Ovarian Cancer: The Added Value of Combining CT with CA-125

**DOI:** 10.3390/diagnostics16213465

**Published:** 2026-10-23

**Authors:** Furkan Akın, Caner Çakır

**Affiliations:** 1 Department of Obstetrics and Gynecology, Ankara Etlik City Hospital, University of Health Sciences, 06170 Ankara, Türkiye; 2 Department of Gynecologic Oncology, Ankara Etlik City Hospital, University of Health Sciences, 06170 Ankara, Türkiye

**Keywords:** ovarian neoplasms, lymphatic metastasis, computed tomography, CA-125 antigen, diagnostic accuracy, lymphadenectomy

## Abstract

**Background/Objectives:** Preoperative prediction of retroperitoneal lymph node (LN) metastasis in epithelial ovarian cancer (EOC) may inform surgical planning and counselling. We evaluated the diagnostic performance of contrast-enhanced computed tomography (CT) for pathological LN metastasis and explored whether adding serum CA-125 to CT findings improves prediction. **Methods:** This single-centre retrospective diagnostic accuracy study included 97 patients with invasive EOC who underwent primary (*n* = 81) or interval (*n* = 16) cytoreductive surgery with histopathological LN assessment (2022–2025) and CT within two months before surgery. Pelvic and para-aortic CT findings were compared with histopathology at the patient and regional levels. A logistic model combining these CT findings with log_2_-transformed CA-125 was compared with the CT-only model using a likelihood-ratio test and was internally validated by bootstrapping. **Results:** Pathological LN metastasis was present in 31 patients (32.0%). At the patient level, CT had a sensitivity of 58% and a specificity of 67%; a short-axis threshold of >10 mm increased specificity to 94% (sensitivity 23%). Adding CA-125 to the CT model improved model fit (likelihood-ratio *p* = 0.019) and discrimination (optimism-corrected area under the curve 0.63 vs. 0.70), with acceptable calibration (corrected slope 0.86). The association of CA-125 with LN metastasis was attenuated after adjustment for high-grade serous histology (adjusted odds ratio per doubling 1.18, 95% confidence interval 0.98–1.43) and was not evident in patients with stage III–IV disease. **Conclusions:** CT has limited sensitivity for LN metastasis in EOC, although enlarged nodes are highly specific. CA-125 may add information to CT findings, but this largely reflects differences in disease extent and histology. These preliminary findings require prospective, externally validated studies before any clinical application.

## 1. Introduction

Ovarian cancer remains one of the most lethal gynaecological malignancies worldwide, largely because most patients present with advanced-stage disease [1]. Retroperitoneal lymph node (LN) involvement is a key prognostic factor and an integral component of the International Federation of Gynecology and Obstetrics (FIGO) staging system, in which isolated retroperitoneal nodal metastasis defines stage IIIA1 disease [2,3]. Knowledge of nodal status therefore affects staging, prognostic counselling and treatment planning.

The role of systematic lymphadenectomy has changed substantially in recent years. In apparent early-stage disease, pelvic and para-aortic lymphadenectomy remains part of comprehensive surgical staging for most histological types [4]. In advanced disease, however, the Lymphadenectomy in Ovarian Neoplasms (LION) trial showed that systematic lymphadenectomy in patients with macroscopically complete resection and clinically negative nodes did not improve overall or progression-free survival but increased postoperative morbidity—even though occult nodal metastases were found in 55.7% of patients in the lymphadenectomy arm [5]. Current European recommendations therefore advise against systematic lymphadenectomy in patients with complete intra-abdominal resection whose nodes are non-suspicious on both preoperative imaging and intraoperative evaluation, whereas enlarged or suspicious nodes should be removed [4]. Preoperative imaging thus contributes directly to the definition of clinically negative nodes. Preoperative information on nodal status may also assist counselling and the planning of retroperitoneal assessment, although predicting nodal involvement does not in itself establish a benefit from removing involved nodes.

Contrast-enhanced computed tomography (CT) is the standard imaging modality for preoperative staging, but it assesses nodes mainly on the basis of size and morphology, so that normal-sized nodes harbouring metastases are missed. In a recent meta-analysis, the pooled sensitivity and specificity of CT for pelvic and para-aortic nodal metastasis in epithelial ovarian cancer were 47% and 99%, respectively, compared with 81% and 96% for positron emission tomography (PET)/CT, although the CT estimates were based on only four studies [6]. Single-centre series based on routine CT reports have reported sensitivities of 41–62% with specificities of 52–89% [7,8,9]. Serum CA-125 has also been associated with nodal metastasis, but with limited discrimination and specificity at any single threshold [8,10,11,12], and combining imaging with CA-125 has been reported to increase sensitivity [10]. Prediction models combining CT-reported nodal status, clinical factors, serum markers or radiomic features have been proposed [13,14,15,16,17], yet few studies have assessed the incremental value of CA-125 over CT findings using region-matched histopathology as the reference standard.

The aims of this study were (i) to determine the diagnostic accuracy of preoperative contrast-enhanced CT for pathological LN metastasis in invasive epithelial ovarian cancer (EOC), at the patient level and separately for the pelvic and para-aortic regions, and (ii) to explore whether adding CA-125 to CT findings improves the prediction of pathological LN metastasis. The study is reported in accordance with the Standards for Reporting of Diagnostic Accuracy Studies (STARD) 2015 guideline [18].

## 2. Materials and Methods

### 2.1. Study Design, Participants and Data Collection

This single-centre retrospective diagnostic accuracy study was conducted at the Gynaecologic Oncology Clinic of Ankara Etlik City Hospital, a tertiary referral centre. It was approved by the Scientific Research Ethics Committee of Ankara Etlik City Hospital (approval no. AEŞH-BADEK1-2025-055; 7 May 2025). All consecutive patients who underwent surgery for an ovarian malignancy between October 2022 and March 2025 and who had contrast-enhanced CT of the thorax, abdomen and pelvis were identified through the hospital information system.

Patients were eligible if they were aged ≥18 years, had histologically confirmed invasive EOC, had undergone contrast-enhanced CT within two months before surgery, and had at least one lymph node removed and examined histopathologically. Patients with borderline tumours, non-epithelial ovarian tumours (sex cord–stromal or germ cell tumours), metastatic tumours of non-ovarian origin or non-epithelial malignancies were excluded, as were patients operated on for recurrent disease and patients in whom no lymph nodes were removed, because the reference standard was unavailable for them. Patients undergoing interval cytoreductive surgery after neoadjuvant chemotherapy (NACT) were included; in these patients, the CT and laboratory values obtained before surgery, i.e., after NACT, were analysed. The selection of participants is shown in Figure 1. This article is derived from the first author’s medical specialty thesis; for the present analysis, the original thesis dataset was re-analysed using the stricter eligibility criteria described above.

Data were extracted retrospectively by one investigator (F.A.), who compared the preoperative CT report of each patient with the postoperative pathology report. The extractor was therefore not blinded to the reference standard; however, all index-test results were taken from reports issued before surgery.

### 2.2. Index Test

All CT examinations were performed at our institution as part of routine preoperative staging; no external examinations were used. All examinations were performed on a 128-slice CT scanner (Revolution Frontier; GE Healthcare, Chicago, IL, USA). After a single intravenous dose of iodinated contrast medium, scanning was triggered by bolus tracking (SmartPrep); the thorax was scanned first, followed by the abdomen and pelvis in the portal venous phase. Images were acquired at a slice thickness of 0.625 mm, and 2.5 mm axial, coronal and sagittal reformats were used for reporting. CT reports were written by radiologists before surgery, without knowledge of the histopathological results, and images were not re-reviewed for this study. For each nodal region (pelvic, para-aortic and cardiophrenic), the region was coded as radiologically LN-positive when the report described any lymph node with a measurement in that region (for example, “a 7 × 5 mm lymph node is observed”), irrespective of whether the radiologist described the node as suspicious, and the long- and short-axis diameters of the largest reported node were recorded. Regions without a nodal description were coded as radiologically LN-negative. No equivocal or unmeasured nodal descriptions were encountered: every reported node had a recorded short-axis diameter, so the size-based analysis involved no missing measurements. One report contained no explicit statement on lymph nodes and was coded as negative. Morphological features such as nodal shape, loss of the fatty hilum, necrosis or capsular irregularity were not consistently reported and could not be assessed.

Two index-test definitions were used: (i) a reported node of any size (primary, report-based definition) and (ii) an enlarged node with a short-axis diameter >10 mm (secondary, size-based definition). At the patient level, radiological LN status was defined by the pelvic and para-aortic findings only, because cardiophrenic dissection was performed only for suspicious nodes, so that cardiophrenic CT findings lacked a reference standard in most patients; cardiophrenic findings were therefore analysed descriptively.

### 2.3. Surgical Procedure and Reference Standard

In accordance with institutional practice, patients with apparent early-stage disease underwent systematic pelvic and para-aortic lymphadenectomy up to the level of the renal veins. In patients with advanced disease in whom complete cytoreduction was the aim, systematic pelvic and para-aortic lymphadenectomy or resection of suspicious nodes was performed. Cardiophrenic lymph node dissection was performed only when suspicious nodes were present. Lymph nodes were examined with routine haematoxylin–eosin staining and immunohistochemistry. Histopathological examination of the removed nodes served as the reference standard. A patient was considered pathologically LN-positive if at least one metastatic node was found in any region. Regional pathological status was defined analogously, and regional analyses were restricted to patients in whom nodes from the corresponding region had been removed and examined.

### 2.4. Clinical Variables

Age, body mass index, menopausal status and the most recent preoperative serum values of haemoglobin, albumin, CA-125, CA 19-9, CA 15-3, carcinoembryonic antigen (CEA) and alpha-fetoprotein (AFP) were recorded. Surgical stage was assigned according to the FIGO 2014 staging system [2], and histological type was classified according to the World Health Organization classification.

### 2.5. Statistical Analysis

Continuous variables are presented as mean ± standard deviation or median (interquartile range) and were compared using Welch’s *t*-test or the Mann–Whitney U test, as appropriate. Categorical variables are presented as numbers (percentages) and were compared using the chi-square test or, when any expected cell count was <5, Fisher’s exact test. Sensitivity, specificity, positive predictive value (PPV) and negative predictive value (NPV) were calculated with Wilson 95% confidence intervals (CIs), and the area under the receiver operating characteristic curve (AUC) was estimated with DeLong 95% CIs [19].

Diagnostic accuracy was assessed (i) at the patient level in all patients, comparing the radiological pelvic/para-aortic status with the pathological LN status; (ii) in the subset of patients with histopathological assessment of both the pelvic and para-aortic regions, comparing the radiological with the pathological pelvic/para-aortic status, so that the index test and reference standard covered the same anatomical regions; and (iii) separately for each region.

Associations with pathological LN metastasis were assessed using univariable logistic regression; CA-125 and CA 15-3 were log_2_-transformed, so that odds ratios (ORs) refer to a doubling of the serum concentration. Two nested models were prespecified: a CT model including the radiological pelvic and para-aortic status (each coded 1 = reported node, 0 = no reported node) and a combined model adding log_2_ CA-125 to the CT model, giving approximately ten outcome events per predictor. The incremental contribution of CA-125 was assessed with a likelihood-ratio test comparing the two nested models fitted in the same patients. To explore confounding by histology, the combined model was additionally adjusted for high-grade serous histology. Postoperative FIGO stage was not used as a covariate because nodal status contributes to stage assignment.

Internal validation was performed with the bootstrap optimism-correction method using 1000 resamples [20]. In each resample, the complete modelling procedure (estimation of all coefficients; no variable selection was involved) was repeated, and model performance was evaluated both in the bootstrap sample and in the original sample. The mean difference (optimism) was subtracted from the apparent performance to obtain optimism-corrected estimates of the AUC, calibration slope, calibration intercept and Brier score. The 95% CI of the difference in AUC between nested models was obtained from the bootstrap percentile distribution. Calibration was displayed graphically using apparent and bias-corrected locally weighted (lowess) calibration curves, together with observed proportions by quintile of predicted risk [21]. Thresholds identified with the Youden index were considered exploratory. The prediction model is reported in line with the TRIPOD + AI statement (Transparent Reporting of a multivariable prediction model for Individual Prognosis Or Diagnosis + Artificial Intelligence) where applicable [22].

No a priori sample size calculation was performed, and all eligible patients were included. Statistical power and precision were therefore assessed post hoc. First, the precision of sensitivity and specificity was expressed as the half-width of their 95% CIs and compared with the number of patients required for a half-width of 10 percentage points [23]. Second, the power of the likelihood-ratio test for adding CA-125 to the CT model, with and without adjustment for high-grade serous histology, was estimated by simulation (2000 replicates), assuming the observed effect sizes and covariate distribution; the sample size required for 80% power was estimated by resampling the covariates. Third, the minimum sample size for developing the combined model was calculated according to the criteria of Riley et al. [24].

Prespecified sensitivity analyses were restricted to patients with both regions examined, patients undergoing primary cytoreduction and patients with ≥10 examined lymph nodes. Exploratory subgroup analyses were performed in patients with high-grade serous carcinoma and in patients with FIGO stage III–IV disease; the latter must be interpreted with caution because stage is assigned postoperatively and partly reflects nodal status. Two-sided *p* < 0.05 was considered statistically significant. Analyses were performed in Python 3.13 (Python Software Foundation, Wilmington, DE, USA) using SciPy 1.18, statsmodels 0.15, scikit-learn 1.9 and pandas 3.0.

### 2.6. Use of Generative Artificial Intelligence

A generative artificial intelligence (GenAI) tool, Claude (Opus 5.5 model; Anthropic, San Francisco, CA, USA; accessed through the Claude application in October 2026), was used (i) to write and execute the Python code for the statistical analyses described above, according to an analysis plan agreed by the authors and the additional analyses requested during peer review; (ii) to generate the figures; (iii) to draft and language-edit the manuscript text and the response to the reviewers; and (iv) to check the bibliographic details of the references against online databases. Only a de-identified dataset containing no patient names or identification numbers was processed with the tool. Data collection, i.e., the review of the CT and pathology reports and the coding of all variables, was performed manually by the first author without the use of GenAI. All outputs were reviewed by the authors, who take full responsibility for the study design, the interpretation of the results and the content of the manuscript.

## 3. Results

### 3.1. Participants

Of 149 patients screened, 40 did not have invasive EOC, 3 were operated on for recurrent disease, and 9 had no lymph nodes removed, leaving 97 patients for analysis (81 primary and 16 interval cytoreductions; Figure 1). Systematic pelvic and para-aortic lymphadenectomy was performed in 85 patients (87.6%), nodal sampling or removal of bulky nodes in 8, and the nodal procedure was not specified in 4. Nodes from both the pelvic and para-aortic regions were examined in 82 patients. The median number of examined lymph nodes was 19 (interquartile range 11–27), with 11 (7–16) pelvic and 10 (5–13) para-aortic nodes. Pathological LN metastasis was present in 31 patients (32.0%).

Patient characteristics are summarised in Table 1. Compared with pathologically LN-negative patients, pathologically LN-positive patients had higher CA-125 (median 432.0 vs. 138.0 U/mL; *p* = 0.003) and CA 15-3 levels (*p* = 0.036) and lower CA 19-9 levels (*p* = 0.004). Systematic lymphadenectomy was more frequent in pathologically LN-negative patients (*p* = 0.016), whereas the number of examined nodes did not differ (*p* = 0.699).

Stage III–IV disease was present in 59 patients (60.8%), and high-grade serous carcinoma was the predominant histological type (64 patients, 66%; Table 2). In keeping with FIGO staging, all pathologically LN-positive patients had stage III–IV disease. High-grade serous carcinoma accounted for 83.9% of pathologically LN-positive and 57.6% of pathologically LN-negative patients.

### 3.2. CT Findings and Histopathology

Overall, 52 pelvic or para-aortic nodes were reported on CT, with a median short-axis diameter of 8.0 (5.0–10.2) mm; 18 measured ≤5 mm, 21 measured 6–10 mm and 13 measured >10 mm. The relationship between CT findings and histopathology is shown in Table 3. At the patient level, 58.1% of pathologically LN-positive and 33.3% of pathologically LN-negative patients were radiologically LN-positive (*p* = 0.021), and an enlarged node was found in 22.6% and 6.1%, respectively (*p* = 0.034). Findings were similar in the 82 patients with both regions examined. In both the pelvic and para-aortic regions, radiological LN positivity was more frequent among patients with regional metastasis (pelvic *p* = 0.018; para-aortic *p* = 0.022), and reported pelvic nodes were larger in pathologically positive than in negative cases (*p* = 0.016).

Cardiophrenic nodes were reported on CT in 21 patients, but cardiophrenic dissection was performed in only 8 (6 with metastasis); CT had reported cardiophrenic nodes in 3 of these 6 patients and in 1 of the 2 without metastasis. In 10 patients, cardiophrenic nodes were reported without pelvic or para-aortic findings; only 1 of them underwent cardiophrenic dissection. Owing to this limited verification, cardiophrenic findings were not included in the patient-level radiological status.

### 3.3. Diagnostic Accuracy of CT

Diagnostic accuracy estimates are given in Table 4. At the patient level, CT had a sensitivity of 58% (95% CI 41–74) and a specificity of 67% (95% CI 55–77), with an NPV of 77%. Applying the >10 mm short-axis criterion increased specificity to 94% (95% CI 85–98) at the cost of sensitivity (23%). In the 82 patients with both regions examined, sensitivity and specificity for pelvic or para-aortic metastasis were 58% and 67% (NPV 80%), and the size criterion yielded a specificity of 97% (95% CI 88–99) with a PPV of 71%. In the pelvic region, sensitivity and specificity were 38% and 89%; with the size criterion, specificity was 99%, and PPV was 80%. In the para-aortic region, sensitivity and specificity were 57% and 70%, and the size criterion yielded a specificity of 96% with a sensitivity of 13%.

Diagnostic performance varied with disease extent. All 32 patients with stage I–II disease were pathologically LN-negative; 10 of them were radiologically LN-positive (specificity 69%), whereas only 1 had an enlarged node (specificity 97%). Among the 59 patients with stage III–IV disease, sensitivity and specificity were 57% and 62%, respectively (Table S1).

### 3.4. CA-125 and the Combined Model

In univariable analysis, CA-125 (OR per doubling 1.29, 95% CI 1.08–1.54; *p* = 0.005), CA 15-3 (*p* = 0.046), high-grade serous histology (OR 3.83, 95% CI 1.31–11.22; *p* = 0.014) and radiological pelvic (*p* = 0.028) and para-aortic (*p* = 0.015) LN positivity were associated with pathological LN metastasis (Table 5). In the combined model, CA-125 remained associated with LN metastasis after adjustment for regional CT findings (adjusted OR 1.24, 95% CI 1.03–1.49; *p* = 0.022). After additional adjustment for high-grade serous histology, the association was attenuated and no longer statistically significant (adjusted OR 1.18, 95% CI 0.98–1.43; *p* = 0.084).

The combined model is given by logit(p) = −2.953 + 0.837 × pelvic + 0.645 × para-aortic + 0.217 × log_2_(CA-125, U/mL), where pelvic and para-aortic are coded 1 if radiologically LN-positive and 0 otherwise, and p is the predicted probability of pathological LN metastasis. Adding CA-125 to the CT model improved model fit (likelihood-ratio χ^2^ = 5.53, df = 1, *p* = 0.019) and discrimination (apparent AUC 0.65 vs. 0.73; difference 0.09, bootstrap 95% CI 0.02–0.19; optimism-corrected AUC 0.63 vs. 0.70; Table 5, Figure 2). Conversely, adding the CT findings to CA-125 alone did not significantly improve model fit (χ^2^ = 5.18, df = 2, *p* = 0.075; optimism-corrected AUC 0.69 vs. 0.70). The calibration of the combined model was acceptable, with an optimism-corrected calibration slope of 0.86, a calibration intercept of −0.08 and a Brier score of 0.190 (corrected 0.208; Figure S1). In exploratory analyses, the Youden-optimal CA-125 threshold was 142 U/mL (sensitivity 87%, specificity 50%), and the Youden-optimal predicted probability of the combined model was 0.25 (sensitivity 81%, specificity 59%); these thresholds were derived in this cohort and require validation.

### 3.5. Sensitivity and Subgroup Analyses

The improvement in model fit with CA-125 was consistent in patients with both regions examined (outcome: pelvic or para-aortic metastasis; *p* = 0.010), in patients undergoing primary cytoreduction (*p* < 0.001) and in patients with ≥10 examined nodes (*p* = 0.006) (Table S1). In the exploratory subgroup of 64 patients with high-grade serous carcinoma (26 events), the association of CA-125 was in the same direction but weaker and not statistically significant (OR per doubling 1.21, 95% CI 0.99–1.48; likelihood-ratio *p* = 0.102). In patients with stage III–IV disease, CA-125 did not discriminate between pathologically LN-positive and LN-negative patients (AUC 0.56; likelihood-ratio *p* = 0.481).

### 3.6. Statistical Power and Precision

With 31 pathologically LN-positive and 66 LN-negative patients, the 95% CIs of patient-level sensitivity and specificity had half-widths of about 17 and 11 percentage points, respectively; estimating a sensitivity of 58% with a half-width of 10 percentage points would have required 94 LN-positive patients (approximately 295 patients at the observed prevalence of 32%). Assuming the observed effect sizes, the simulated power of the likelihood-ratio test for adding CA-125 to the CT model was 67%, and it was 45% after additional adjustment for high-grade serous histology; 80% power would have required approximately 150 and 250 patients, respectively. According to the criteria of Riley et al., developing a model with three predictor parameters at an outcome prevalence of 32% and a Cox–Snell R^2^ of 0.13 would require at least 190 patients to limit overfitting (target shrinkage factor 0.9) and 335 patients (108 events) to estimate the overall risk precisely [24]. The study was therefore underpowered for the histotype-adjusted analysis and for definitive model development.

## 4. Discussion

In this cohort of patients with invasive EOC and histologically verified nodal status, preoperative contrast-enhanced CT showed limited sensitivity for LN metastasis, whereas enlarged nodes (short axis > 10 mm) were highly specific. Adding CA-125 to the regional CT findings improved model fit and discrimination in this mixed-stage cohort. However, the association of CA-125 with nodal metastasis was attenuated after adjustment for histotype and was not evident in patients with stage III–IV disease, indicating that its apparent added value largely reflects differences in disease extent and histological composition. These findings should therefore be regarded as preliminary.

### 4.1. Diagnostic Performance of CT

At the patient level, CT had a sensitivity of 58% and a specificity of 67%. In the meta-analysis by Mimoun et al., the pooled sensitivity and specificity of CT were 47% and 99% [6]; Widschwendter et al. reported 40.7% and 89.1% [7]; Verma et al. reported 56.5% and 78.9% using a 1 cm or abnormal-morphology criterion [8], and Uysal et al., who also analysed routine reports, reported 62% and 52% [9]. The moderate specificity in our study is largely explained by the report-based definition, in which any lymph node described with a measurement was coded as positive, irrespective of whether the radiologist considered it suspicious; 18 of the 52 reported pelvic or para-aortic nodes measured ≤5 mm. The mere mention of a small node does not imply suspected metastasis, and when the conventional >10 mm short-axis criterion was applied, specificity rose to 94% at the patient level, and 97% in patients with both regions examined, approaching the pooled estimate, whereas sensitivity fell to about one in four. This trade-off reflects the fundamental limitation of size-based nodal assessment: micrometastases in normal-sized nodes cannot be detected. Morphological criteria such as nodal shape, loss of the fatty hilum, necrosis and capsular irregularity may improve the characterisation of non-enlarged nodes, but they were not consistently reported in our routine reports and could not be evaluated; standardised reporting lexicons and structured reports, as recommended for ovarian cancer staging, may address this limitation [4,25]. Clinically, an enlarged pelvic or para-aortic node on CT is highly predictive of metastasis (PPV 71% in patients with both regions examined), whereas a negative CT does not exclude nodal disease (NPV 77–80%).

Region-specific specificity was 89% for pelvic and 70% for para-aortic nodes. Widschwendter et al. found comparable specificities for both regions (91.8% and 93.1%), with a higher sensitivity in the para-aortic region (41.2% vs. 25.6%) [7]. The lower para-aortic specificity in our cohort is consistent with the frequent reporting of small para-aortic nodes. Para-aortic nodes are commonly involved in ovarian cancer, in keeping with lymphatic drainage along the infundibulopelvic ligament. Methodologically, the index test and the reference standard should cover the same anatomical regions. We therefore restricted regional analyses to dissected regions and excluded cardiophrenic findings from the patient-level radiological status: cardiophrenic nodes were reported in 21 patients but dissected in only 8, so that counting unverified cardiophrenic findings as positive would have classified unsampled patients as false positives and underestimated specificity. Cardiophrenic nodes are frequently visible on CT in advanced ovarian cancer—nodes of any size were present in 72% of patients with stage III–IV disease in one series [26]—but they are usually resected only when enlarged and when complete intra-abdominal resection is feasible, and the therapeutic value of their resection remains uncertain [27]. The analysis restricted to patients with both regions examined confirmed the patient-level estimates.

### 4.2. Spectrum of Disease

The cohort included patients with all FIGO stages. Because nodal metastasis defines at least stage IIIA1 disease, all patients with stage I–II disease were pathologically LN-negative; in this group, small reported nodes produced false-positive findings in 10 of 32 patients, whereas enlarged nodes did so in only 1. In stage III–IV disease, CT performed less well (specificity 62%), and CA-125 did not discriminate between pathologically LN-positive and LN-negative patients. Diagnostic estimates therefore depend on the case mix, and pooled estimates from a mixed-stage cohort may not apply to patients with suspected advanced disease. Stage-restricted analyses must, however, be interpreted with caution, because FIGO stage is assigned after surgery and partly reflects nodal status, and preoperative clinical stage was not available in our records.

### 4.3. Interpretation of CA-125 and the Combined Model

CA-125 was associated with pathological LN metastasis in univariable analysis and after adjustment for regional CT findings. Its discrimination as a single marker (AUC 0.69) was similar to the AUCs of 0.636, 0.671 and 0.702 reported by Sudolmuş et al., Zhang et al. and Verma et al., respectively [8,11,12], and published optimal thresholds range widely, from about 72 to 1360 U/mL [8,10,11]; the threshold of 142 U/mL identified in our cohort should be regarded as exploratory. Importantly, the association was attenuated after adjustment for high-grade serous histology, which accounted for 83.9% of pathologically LN-positive but only 57.6% of LN-negative patients, and it was weaker and not statistically significant within the high-grade serous subgroup. Together with the absence of discrimination in stage III–IV disease, this suggests that CA-125 acts largely as a marker of tumour burden and histotype rather than as a specific marker of nodal spread. We therefore do not consider CA-125 an independent predictor of nodal metastasis. Recent prediction models have likewise been inconsistent: CA-125 was not retained as an independent predictor in a CT radiomics study and did not differ by nodal status in an ultrasound-based model [16,28], whereas it contributed to an ultrasound radiomics nomogram in high-grade serous carcinoma [17].

In a direct comparison of nested models fitted in the same patients, adding CA-125 to the CT model improved model fit and increased the optimism-corrected AUC from 0.63 to 0.70, whereas adding the CT findings to CA-125 did not significantly improve the model. This pattern is consistent with Kim et al., who reported that combining imaging with preoperative CA-125 increased sensitivity, whereas imaging alone provided the highest specificity [10], and with the nomogram of Xiang et al., which combined CT-reported nodal status with clinical factors and achieved an AUC of 0.775 [13]. CT-based radiomics models have achieved AUCs of 0.84–0.87 in internal test sets, and in one of them the radiologist’s CT report alone yielded AUCs of 0.69–0.72, similar to that of our CT model [14,16]. However, a radiological score for nodal metastasis performed considerably worse in an external validation sample than in its derivation sample [15], underscoring the need for external validation. The calibration slope of 0.86 indicates modest overfitting, with slightly too extreme predictions, and the overall discrimination of the model was moderate. Given these characteristics, a sample size below that recommended for model development [24] and the absence of external validation, the model should be considered exploratory and is not suitable for clinical use.

The paradoxically lower CA 19-9 levels in pathologically LN-positive patients are also best explained by histological composition. High-grade serous carcinoma, which predominated among LN-positive patients, was associated with lower CA 19-9 levels than other histological types (median 8.5 vs. 19.6 U/mL; *p* < 0.001), whereas none of the 5 patients with mucinous carcinoma—typically associated with CA 19-9 elevation—had nodal metastases.

### 4.4. Clinical Implications

The intended population for the approach evaluated here is patients with EOC scheduled for primary or interval cytoreductive surgery, and the target condition is pathological pelvic or para-aortic nodal metastasis. Preoperative estimation of this risk may inform counselling and the planning of retroperitoneal assessment. Predicting nodal involvement must, however, be distinguished from demonstrating a benefit from removing involved nodes. The LION trial showed that systematic lymphadenectomy did not improve survival in advanced disease with complete resection and clinically negative nodes, despite occult metastases in more than half of the patients [5], and current recommendations advise against systematic lymphadenectomy in this setting [4]; our findings therefore do not support using CT or CA-125 to select patients for lymphadenectomy in advanced disease. Conversely, in apparent early-stage disease, in which lymphadenectomy remains part of surgical staging [4], neither a negative CT nor a low CA-125 level should be used to justify omitting nodal assessment. Before any clinical application, the combined approach requires prospective validation in an independent cohort and evaluation of its effect on clinical decisions.

### 4.5. Strengths and Limitations

This study has several strengths. Histopathology of resected nodes served as the reference standard in every patient; most patients underwent systematic pelvic and para-aortic lymphadenectomy; the index test and reference standard were matched by region; the cohort was limited to invasive EOC, the models were prespecified and internally validated, and the results were examined in several sensitivity and subgroup analyses. Several limitations must also be acknowledged. First, the retrospective single-centre design limits generalisability, and the number of events (31) resulted in wide confidence intervals; the post hoc power analysis showed that the study was underpowered for the histotype-adjusted analysis and that the sample size was below that recommended for developing a prediction model [24]. Second, CT findings were extracted from routine reports without central re-review, standardised structured reporting or assessment of morphological features, and inter-observer variability could not be assessed; the report-based definition included small nodes that were not necessarily considered suspicious. Third, data were extracted by one investigator who was not blinded to histopathology, although index-test results were documented before surgery. Fourth, the cohort was heterogeneous with respect to stage and treatment sequence, including 16 patients assessed after NACT; postoperative stage partly reflects nodal status, which limits stage-restricted analyses. Fifth, nodal sampling rather than systematic lymphadenectomy was performed in some patients; patients without nodal examination were excluded, and cardiophrenic findings could rarely be verified, so that partial verification bias cannot be excluded. Sixth, the association of CA-125 may be confounded by histotype and tumour burden. Finally, the model was not externally validated, and no comparison with PET/CT or diffusion-weighted magnetic resonance imaging was possible; PET/CT showed higher pooled accuracy than CT in a meta-analysis [6], although its sensitivity for nodal metastasis was low in a cohort with advanced disease [29].

## 5. Conclusions

In patients with invasive EOC, preoperative contrast-enhanced CT has limited sensitivity for pathological LN metastasis, whereas enlarged pelvic or para-aortic nodes are highly specific. In this retrospective mixed-stage cohort, adding CA-125 to regional CT findings improved prediction, but this association was largely explained by disease extent and histotype. These findings are preliminary and do not establish a diagnostic role for CA-125 in predicting nodal metastasis. Prospective, externally validated studies with standardised imaging criteria are required before CT and CA-125 can be combined to support clinical decisions.

## Figures and Tables

**Figure 1:**
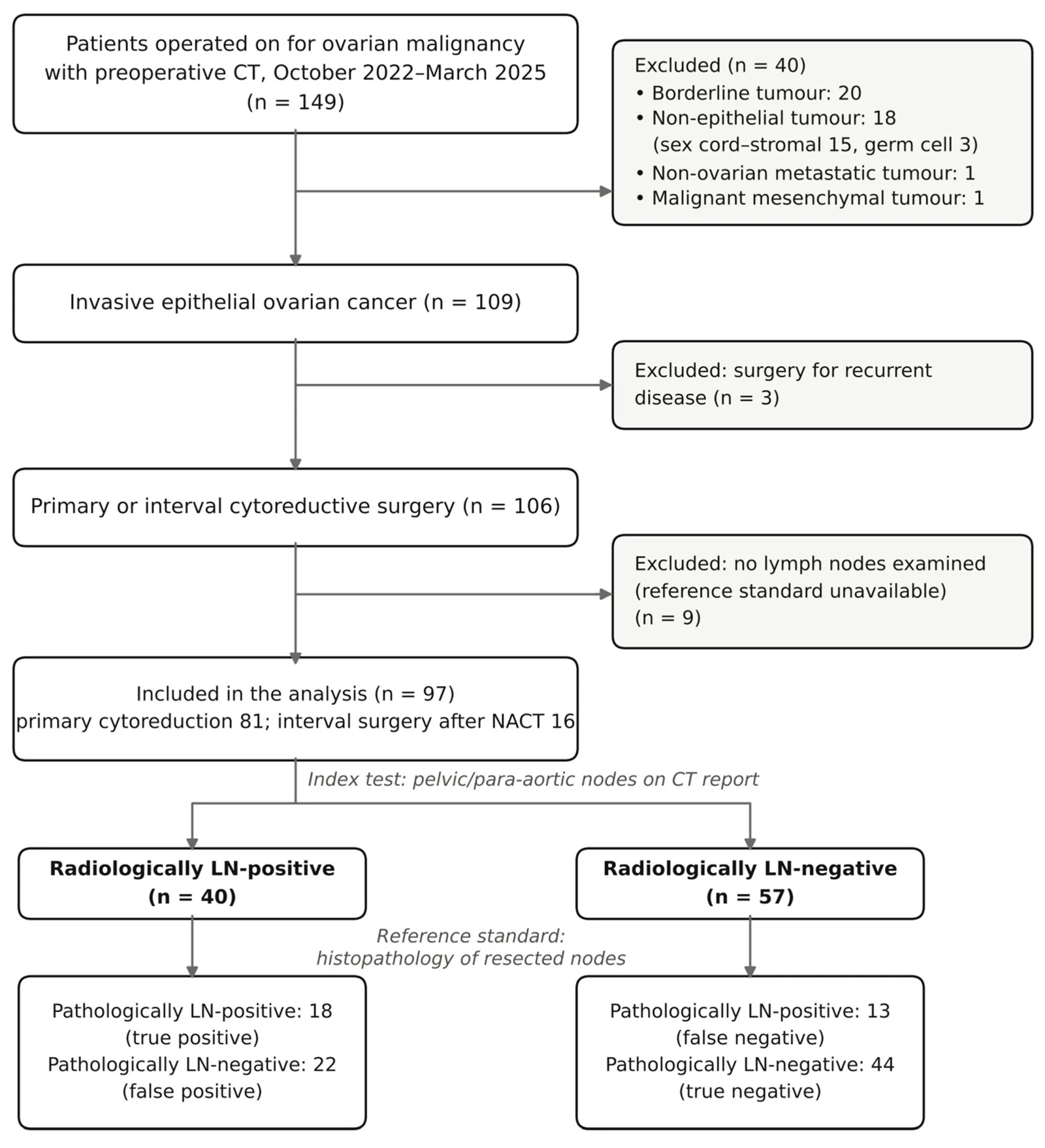
STARD flow diagram of participant selection and of index-test results (pelvic and para-aortic CT findings) by reference-standard results. CT, computed tomography; EOC, epithelial ovarian cancer; LN, lymph node; NACT, neoadjuvant chemotherapy; STARD, Standards for Reporting of Diagnostic Accuracy Studies.

**Figure 2:**
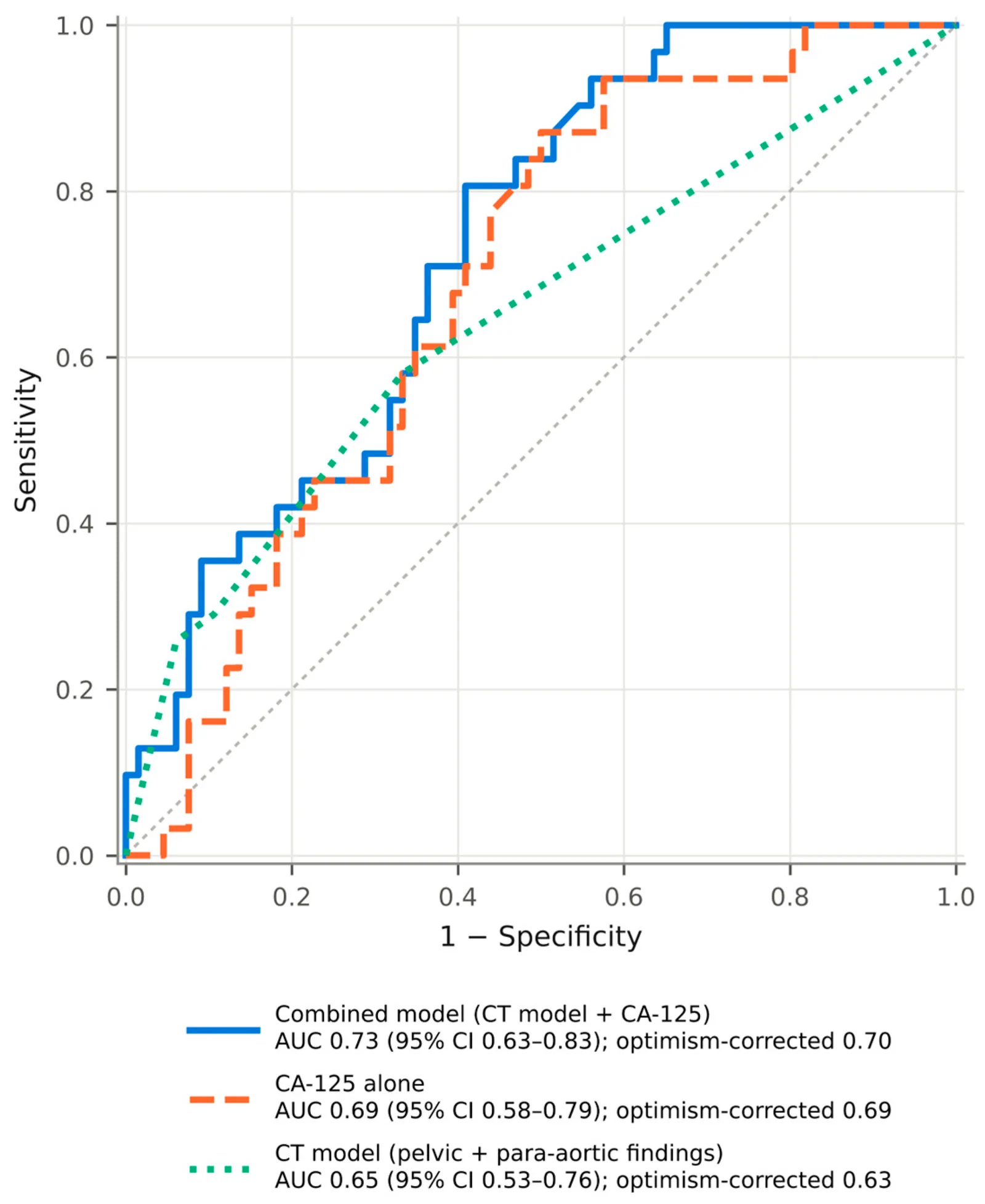
Receiver operating characteristic curves of the CT model (radiological pelvic and para-aortic status), CA-125 alone and the combined model (CT model + log_2_ CA-125), with apparent and optimism-corrected areas under the curve (AUC). CI, confidence interval; CT, computed tomography.

**Table 1:** Clinical characteristics and serum markers according to pathological lymph node status.

Characteristic	All (*n* = 97)	Pathologically LN-Negative (*n* = 66)	Pathologically LN-Positive (*n* = 31)	*p*
Age, years	56.4 ± 11.0	57.3 ± 11.6	54.5 ± 9.4	0.214
Body mass index, kg/m^2^	28.4 ± 5.5	28.9 ± 5.6	27.5 ± 5.3	0.248
Postmenopausal, *n* (%)	75 (77.3)	50 (75.8)	25 (80.6)	0.592
Hemoglobin, g/dL	12.7 (11.4–13.5)	12.6 (11.4–13.5)	13.0 (11.8–13.4)	0.612
Albumin, g/L	38.8 (33.9–42.5)	39.6 (34.0–43.2)	36.4 (34.0–41.2)	0.109
CA-125, U/mL	200.0 (54.7–1017.0)	138.0 (38.3–567.5)	432.0 (177.5–1531.0)	0.003
CA 15-3, U/mL	34.2 (20.6–80.7)	28.6 (18.6–56.9)	60.0 (21.9–233.0)	0.036
CA 19-9, U/mL	10.6 (4.4–39.8)	13.8 (6.4–48.0)	7.7 (2.8–11.2)	0.004
CEA, ng/mL	1.4 (0.8–2.2)	1.5 (0.9–2.4)	1.0 (0.7–1.9)	0.119
AFP, ng/mL	2.0 (1.5–2.8)	2.1 (1.6–2.8)	2.0 (1.1–2.7)	0.403
Interval surgery after NACT, *n* (%)	16 (16.5)	11 (16.7)	5 (16.1)	0.947
Systematic pelvic and para-aortic lymphadenectomy, n (%)	85 (87.6)	62 (93.9)	23 (74.2)	0.016
Lymph nodes examined, *n*	19 (11–27)	18 (11–27)	20 (11–26)	0.699

Values are mean ± standard deviation, median (interquartile range) or *n* (%). *p* values from Welch’s *t*-test (age, body mass index), the Mann–Whitney U test (other continuous variables) or the chi-square/Fisher’s exact test (categorical variables). AFP, alpha-fetoprotein; CEA, carcinoembryonic antigen; LN, lymph node; NACT, neoadjuvant chemotherapy.

**Table 2:** FIGO stage and histological type according to pathological lymph node status.

Category	All (*n* = 97)	Pathologically LN-Negative (*n* = 66)	Pathologically LN-Positive (*n* = 31)
FIGO 2014 stage			
Stage I	27 (27.8)	27 (40.9)	0 (0.0)
Stage II	5 (5.2)	5 (7.6)	0 (0.0)
Stage III	26 (26.8)	13 (19.7)	13 (41.9)
Stage IV	33 (34.0)	16 (24.2)	17 (54.8)
Not available	6 (6.2)	5 (7.6)	1 (3.2)
Histological type			
High-grade serous carcinoma	64 (66.0)	38 (57.6)	26 (83.9)
Clear cell carcinoma	12 (12.4)	10 (15.2)	2 (6.5)
Endometrioid carcinoma	8 (8.2)	8 (12.1)	0 (0.0)
Low-grade serous carcinoma	6 (6.2)	4 (6.1)	2 (6.5)
Mucinous carcinoma	5 (5.2)	5 (7.6)	0 (0.0)
Other epithelial carcinoma	2 (2.1)	1 (1.5)	1 (3.2)

Values are *n* (%). Stage according to FIGO 2014; stage was not available in the records of 6 patients. “Other epithelial carcinoma” comprises one malignant Brenner tumour and one malignant epithelial tumour not otherwise specified. FIGO, International Federation of Gynecology and Obstetrics; LN, lymph node.

**Table 3:** CT nodal findings according to histopathological lymph node status, at the patient level and by region.

CT Parameter	Pathologically LN-Negative	Pathologically LN-Positive	*p*
Patient level: pelvic or para-aortic CT findings vs. any nodal metastasis (*n* = 97; 66 pathologically LN-negative/31 LN-positive)			
Radiologically LN-positive (reported node), *n* (%)	22 (33.3)	18 (58.1)	0.021 ^a^
Enlarged node (short axis > 10 mm), *n* (%)	4 (6.1)	7 (22.6)	0.034 ^b^
Both regions examined: pelvic or para-aortic CT findings vs. pelvic or para-aortic metastasis (*n* = 82; 58/24)			
Radiologically LN-positive (reported node), *n* (%)	19 (32.8)	14 (58.3)	0.032 ^a^
Enlarged node (short axis > 10 mm), *n* (%)	2 (3.4)	5 (20.8)	0.021 ^b^
Pelvic region (*n* = 88; 72/16)			
Radiologically LN-positive (reported node), *n* (%)	8 (11.1)	6 (37.5)	0.018 ^b^
Enlarged node (short axis > 10 mm), *n* (%)	1 (1.4)	4 (25.0)	0.003 ^b^
Short axis of reported nodes, mm (*n* = 8/6)	6.5 (4.8–8.5)	12.5 (9.0–14.5)	0.016 ^c^
Para-aortic region (*n* = 90; 67/23)			
Radiologically LN-positive (reported node), *n* (%)	20 (29.9)	13 (56.5)	0.022 ^a^
Enlarged node (short axis > 10 mm), *n* (%)	3 (4.5)	3 (13.0)	0.171 ^b^
Short axis of reported nodes, mm (*n* = 20/13)	5.5 (5.0–9.0)	10.0 (5.0–10.0)	0.156 ^c^

Values are *n* (%) or median (interquartile range). Radiologically LN-positive: any lymph node described with a measurement in the CT report. Regional analyses include only patients in whom nodes from that region were removed; short-axis comparisons include only reported nodes. ^a^ Chi-square test; ^b^ Fisher’s exact test; ^c^ Mann–Whitney U test. CT, computed tomography; LN, lymph node.

**Table 4:** Diagnostic accuracy of contrast-enhanced CT for lymph node metastasis.

CT Criterion	TP/FP/FN/TN	Sensitivity, %	Specificity, %	PPV, %	NPV, %	AUC
Patient level (*n* = 97)						
Reported node	18/22/13/44	58 (41–74)	67 (55–77)	45 (31–60)	77 (65–86)	0.62 (0.52–0.73)
Short axis > 10 mm	7/4/24/62	23 (11–40)	94 (85–98)	64 (35–85)	72 (62–80)	0.58 (0.50–0.66)
Both regions examined (*n* = 82)						
Reported node	14/19/10/39	58 (39–76)	67 (54–78)	42 (27–59)	80 (66–89)	0.63 (0.51–0.75)
Short axis > 10 mm	5/2/19/56	21 (9–40)	97 (88–99)	71 (36–92)	75 (64–83)	0.59 (0.50–0.67)
Pelvic region (*n* = 88)						
Reported node	6/8/10/64	38 (18–61)	89 (80–94)	43 (21–67)	86 (77–92)	0.63 (0.50–0.76)
Short axis > 10 mm	4/1/12/71	25 (10–49)	99 (93–100)	80 (38–96)	86 (76–92)	0.62 (0.51–0.73)
Para-aortic region (*n* = 90)						
Reported node	13/20/10/47	57 (37–74)	70 (58–80)	39 (25–56)	82 (71–90)	0.63 (0.52–0.75)
Short axis > 10 mm	3/3/20/64	13 (5–32)	96 (88–98)	50 (19–81)	76 (66–84)	0.54 (0.47–0.62)

Patient level: radiological pelvic/para-aortic status vs. pathological LN status in any region. Both regions examined: radiological vs. pathological pelvic/para-aortic status in patients with histopathological assessment of both regions. Reported node: any lymph node described with a measurement in the CT report; short axis > 10 mm: enlarged node. Values in parentheses are 95% confidence intervals (Wilson method for proportions; DeLong method for AUC). AUC, area under the receiver operating characteristic curve; FN, false negative; FP, false positive; NPV, negative predictive value; PPV, positive predictive value; TN, true negative; TP, true positive.

**Table 5:** Predictors of pathological lymph node metastasis and discrimination of nested models.

Variable	Univariable OR (95% CI)	*p*	Combined Model aOR (95% CI) ^a^	*p*	Histology-Adjusted aOR (95% CI) ^b^	*p*
(**A**) Logistic regression (outcome: pathological lymph node metastasis)						
Age (per year)	0.98 (0.94–1.02)	0.247	—	—	—	—
Postmenopausal status	1.33 (0.46–3.83)	0.593	—	—	—	—
CA-125 (per doubling)	1.29 (1.08–1.54)	0.005	1.24 (1.03–1.49)	0.022	1.18 (0.98–1.43)	0.084
CA 15-3 (per doubling)	1.28 (1.00–1.64)	0.046	—	—	—	—
Albumin (per g/L)	0.95 (0.89–1.01)	0.107	—	—	—	—
Interval surgery after NACT	0.96 (0.30–3.05)	0.947	—	—	—	—
High-grade serous histology	3.83 (1.31–11.22)	0.014	—	—	2.58 (0.80–8.28)	0.111
Radiologically LN-positive, pelvic or para-aortic	2.77 (1.15–6.66)	0.023	—	—	—	—
Radiologically LN-positive, pelvic	3.45 (1.14–10.38)	0.028	2.31 (0.68–7.89)	0.182	1.97 (0.57–6.75)	0.283
Radiologically LN-positive, para-aortic	3.00 (1.24–7.28)	0.015	1.91 (0.71–5.13)	0.202	2.04 (0.75–5.57)	0.162
(**B**) Discrimination and comparison of nested models						
Model	Apparent AUC (95% CI)		Optimism-corrected AUC		Likelihood-ratio test	
CT model (pelvic + para-aortic findings)	0.65 (0.53–0.76)		0.63		—	
CA-125 alone	0.69 (0.58–0.79)		0.69		—	
Combined model (CT model + CA-125)	0.73 (0.63–0.83)		0.70		vs. CT model: χ^2^ = 5.53, df = 1, *p* = 0.019; vs. CA-125 alone: χ^2^ = 5.18, df = 2, *p* = 0.075	

^a^ Combined model: radiological pelvic and para-aortic status plus log_2_ CA-125. ^b^ Combined model additionally adjusted for high-grade serous histology. Panel (**B**): all models fitted in the same 97 patients; optimism-corrected AUCs from 1000 bootstrap resamples; difference in AUC between the combined model and the CT model 0.09 (bootstrap 95% CI 0.02–0.19). aOR, adjusted odds ratio; AUC, area under the receiver operating characteristic curve; CI, confidence interval; NACT, neoadjuvant chemotherapy; OR, odds ratio.

## Data Availability

The data presented in this study are available on reasonable request from the corresponding author. The data are not publicly available owing to patient privacy restrictions.

## Supplementary Materials

The following supporting information can be downloaded at: https://www.mdpi.com/article/10.3390/diagnostics16213465/s1, Table S1: Sensitivity and subgroup analyses; Figure S1: Calibration plot of the combined model; Table S2: STARD 2015 checklist. Reference [18] is cited in the Supplementary Materials.

## Abbreviations

AFP, alpha-fetoprotein; aOR, adjusted odds ratio; AUC, area under the receiver operating characteristic curve; CEA, carcinoembryonic antigen; CI, confidence interval; CT, computed tomography; EOC, epithelial ovarian cancer; FIGO, International Federation of Gynecology and Obstetrics; GenAI, generative artificial intelligence; LION, Lymphadenectomy in Ovarian Neoplasms; LN, lymph node; NACT, neoadjuvant chemotherapy; NPV, negative predictive value; OR, odds ratio; PET, positron emission tomography; PPV, positive predictive value; STARD, Standards for Reporting of Diagnostic Accuracy Studies; TRIPOD, Transparent Reporting of a multivariable prediction model for Individual Prognosis Or Diagnosis.

## References

[1] Bray F., Laversanne M., Sung H., Ferlay J., Siegel R.L., Soerjomataram I., Jemal A. (2024). Global cancer statistics 2022: GLOBOCAN estimates of incidence and mortality worldwide for 36 cancers in 185 countries. CA Cancer J. Clin..

[2] Prat J., FIGO Committee on Gynecologic Oncology (2014). Staging classification for cancer of the ovary, fallopian tube, and peritoneum. Int. J. Gynaecol. Obstet..

[3] Berek J.S., Renz M., Kehoe S., Kumar L., Friedlander M. (2021). Cancer of the ovary, fallopian tube, and peritoneum: 2021 update. Int. J. Gynaecol. Obstet..

[4] Ledermann J.A., Matias-Guiu X., Amant F., Concin N., Davidson B., Fotopoulou C., González-Martin A., Gourley C., Leary A., Lorusso D. (2024). ESGO–ESMO–ESP consensus conference recommendations on ovarian cancer: Pathology and molecular biology and early, advanced and recurrent disease. Ann. Oncol..

[5] Harter P., Sehouli J., Lorusso D., Reuss A., Vergote I., Marth C., Kim J.-W., Raspagliesi F., Lampe B., Aletti G. (2019). A randomized trial of lymphadenectomy in patients with advanced ovarian neoplasms. N. Engl. J. Med..

[6] Mimoun C., Rouzier R., Benifla J.L., Fauconnier A., Huchon C. (2021). Preoperative CT or PET/CT to assess pelvic and para-aortic lymph node status in epithelial ovarian cancer? A systematic review and meta-analysis. Diagnostics.

[7] Widschwendter P., Blersch A., Friedl T.W.P., Janni W., Kloth C., de Gregorio A., de Gregorio N. (2020). CT scan in the prediction of lymph node involvement in ovarian cancer—A retrospective analysis of a tertiary gyneco-oncological unit. Geburtshilfe Frauenheilkd..

[8] Verma P., Bahadur A., Rajaram S., Seenivasagam R.K., Chaturvedi J., Mundhra R., Gaurav A., Rao S., Sahoo I., Heda A. (2024). Lymph node evaluation and nodal metastasis prediction in epithelial ovarian cancers: A retrospective study. Turk. J. Obstet. Gynecol..

[9] Uysal N.E., Bakir M.S., Birge Ö., Karadağ C., Şimşek T. (2021). Prediction of lymph node involvement in epithelial ovarian cancer by PET/CT, CT and MRI imaging. Eur. J. Gynaecol. Oncol..

[10] Kim H.S., Park N.H., Chung H.H., Kim J.W., Song Y.S., Kang S.B. (2008). Significance of preoperative serum CA-125 levels in the prediction of lymph node metastasis in epithelial ovarian cancer. Acta Obstet. Gynecol. Scand..

[11] Sudolmuş S., Köroğlu N., Yıldırım G., Ülker V., Gülkılık A., Dansuk R. (2014). Can CA-125 predict lymph node metastasis in epithelial ovarian cancers in Turkish population?. Dis. Markers.

[12] Zhang L., Guan Z., Yin Y., Ou C., Qian H., Tang M., Shen A. (2022). Predictive value of indicator of CA125 combined with D-dimer (ICD) for lymph node metastasis in patients with ovarian cancer: A two center cohort study. J. Cancer.

[13] Xiang H., Yang F., Zheng X., Pan B., Ju M., Xu S., Zheng M. (2023). A nomogram for preoperative prediction of the risk of lymph node metastasis in patients with epithelial ovarian cancer. Curr. Oncol..

[14] Chen H., Wang X., Zhao F., Chen X., Li X., Ning G., Guo Y.-K. (2021). The development and validation of a CT-based radiomics nomogram to preoperatively predict lymph node metastasis in high-grade serous ovarian cancer. Front. Oncol..

[15] Crestani A., Huchon C., Mezzadri M., Marchand E., Place V., Cornelis F., Touboul C., Haddad B., Dabi Y., Benifla J.-L. (2022). A pre-operative radiological score to predict lymph node metastasis in advanced epithelial ovarian cancer. J. Gynecol. Obstet. Hum. Reprod..

[16] Zhang J., Li Q., Liang H., Wang Y., Sun L., Zhang Q., Gao C. (2025). Preoperative prediction of lymph node metastasis in patients with ovarian cancer using contrast-enhanced computed tomography-based intratumoral and peritumoral radiomics features. Front. Oncol..

[17] Qi Y., Liu J., Wang X., Zhang Y., Li Z., Qi X., Huang Y. (2024). Development and validation of an ultrasound-based radiomics nomogram to predict lymph node status in patients with high-grade serous ovarian cancer: A retrospective analysis. J. Ovarian Res..

[18] Bossuyt P.M., Reitsma J.B., Bruns D.E., Gatsonis C.A., Glasziou P.P., Irwig L., Lijmer J.G., Moher D., Rennie D., de Vet H.C.W. (2015). STARD 2015: An updated list of essential items for reporting diagnostic accuracy studies. BMJ.

[19] DeLong E.R., DeLong D.M., Clarke-Pearson D.L. (1988). Comparing the areas under two or more correlated receiver operating characteristic curves: A nonparametric approach. Biometrics.

[20] Collins G.S., Dhiman P., Ma J., Schlussel M.M., Archer L., Van Calster B., Harrell F.E., Martin G.P., Moons K.G.M., van Smeden M. (2024). Evaluation of clinical prediction models (part 1): From development to external validation. BMJ.

[21] Riley R.D., Archer L., Snell K.I.E., Ensor J., Dhiman P., Martin G.P., Bonnett L.J., Collins G.S. (2024). Evaluation of clinical prediction models (part 2): How to undertake an external validation study. BMJ.

[22] Collins G.S., Moons K.G.M., Dhiman P., Riley R.D., Beam A.L., Van Calster B., Ghassemi M., Liu X., Reitsma J.B., van Smeden M. (2024). TRIPOD+AI statement: Updated guidance for reporting clinical prediction models that use regression or machine learning methods. BMJ.

[23] Akoğlu H. (2022). User’s guide to sample size estimation in diagnostic accuracy studies. Turk. J. Emerg. Med..

[24] Riley R.D., Ensor J., Snell K.I.E., Harrell F.E., Martin G.P., Reitsma J.B., Moons K.G.M., Collins G., van Smeden M. (2020). Calculating the sample size required for developing a clinical prediction model. BMJ.

[25] Shinagare A.B., Sadowski E.A., Park H., Brook O.R., Forstner R., Wallace S.K., Horowitz J.M., Horowitz N., Javitt M., Jha P. (2022). Ovarian cancer reporting lexicon for computed tomography (CT) and magnetic resonance (MR) imaging developed by the SAR Uterine and Ovarian Cancer Disease-Focused Panel and the ESUR Female Pelvic Imaging Working Group. Eur. Radiol..

[26] Song S., Chen H., Ning G., Guo Y., Li X. (2023). Clinical significance of enlarged cardiophrenic lymph nodes by CT in advanced ovarian cancer. Front. Oncol..

[27] Agusti N., Bonaldo G., Kahn R.M., Rosati A., Nachira D., Pan T.L., Mburu A., Kochiashvili G., Paredes P., Hsu H.-C. (2024). Cardiophrenic lymph nodes in advanced ovarian cancer. Int. J. Gynecol. Cancer.

[28] Yang Y., Ye X., Zhou B., Liu Y., Feng M., Lv W., Lu D., Cui X., Liu J. (2023). Nomogram for predicting lymph node metastasis in patients with ovarian cancer using ultrasonography: A multicenter retrospective study. BMC Cancer.

[29] Tardieu A., Ouldamer L., Margueritte F., Rossard L., Lacorre A., Bourdel N., Lades G., Sallée C., Monteil J., Gauthier T. (2021). Assessment of lymph node involvement with PET-CT in advanced epithelial ovarian cancer. A FRANCOGYN group study. J. Clin. Med..

