# Supplementary material for: Diagnostic Performance of Contrast-Enhanced Computed Tomography in Predicting Lymph Node Metastasis in Epithelial Ovarian Cancer: The Added Value of Combining CT with CA-125
Source: Diagnostics (Basel). 2026 Oct 23;16(21):3465. doi: 10.3390/diagnostics16213465 (PMC13761751; doi:10.3390/diagnostics16213465)
Supplement: Supplementary file 1 [file diagnostics-16-03465-s001.zip › diagnostics-4651220-supplementary.pdf]

Supplementary Materials

Diagnostic Performance of Contrast-Enhanced Computed Tomography in Predicting Lymph Node Metastasis in Epithelial Ovarian Cancer: The Added Value of Combining CT with CA-125

Table S1. Sensitivity and subgroup analyses.

Analysis	n (events; %)	Reported node: sensitivity, %	Reported node: specificity, %	Short axis >10 mm: sensitivity, %	Short axis >10 mm: specificity, %	CA-125 per doubling, OR (95% CI)	CA-125 per doubling, aOR in combined model (95% CI)	AUC, CT model (apparent / corrected)	AUC, combined model (apparent / corrected)	Likelihood-ratio test, CA-125 added to CT model
Main cohort	97 (31; 32%)	58 (41–74)	67 (55–77)	23 (11–40)	94 (85–98)	1.29 (1.08–1.54); p = 0.005	1.24 (1.03–1.49); p = 0.022	0.65 / 0.63	0.73 / 0.70	χ^2 = 5.53; p = 0.019
Both regions examined; outcome: pelvic or para-aortic metastasis	82 (24; 29%)	58 (39–76)	67 (54–78)	21 (9–40)	97 (88–99)	1.39 (1.12–1.73); p = 0.003	1.34 (1.06–1.69); p = 0.014	0.66 / 0.64	0.77 / 0.74	χ^2 = 6.57; p = 0.010
Primary cytoreduction only	81 (26; 32%)	50 (32–68)	65 (52–77)	15 (6–34)	95 (85–98)	1.50 (1.19–1.89); p < 0.001	1.45 (1.14–1.85); p = 0.002	0.61 / 0.58	0.78 / 0.75	χ^2 = 11.11; p < 0.001
≥10 lymph nodes examined	80 (25; 31%)	56 (37–73)	65 (52–77)	20 (9–39)	95 (85–98)	1.42 (1.14–1.79); p = 0.002	1.38 (1.08–1.76); p = 0.009	0.63 / 0.60	0.76 / 0.72	χ^2 = 7.65; p = 0.006
High-grade serous carcinoma only	64 (26; 41%)	62 (43–78)	63 (47–77)	27 (14–46)	89 (76–96)	1.21 (0.99–1.48); p = 0.059	1.19 (0.96–1.46); p = 0.110	0.66 / 0.63	0.70 / 0.66	χ^2 = 2.67; p = 0.102
FIGO stage III–IV only	59 (30; 51%)	57 (39–73)	62 (44–77)	23 (12–41)	90 (74–96)	1.10 (0.90–1.35); p = 0.353	1.08 (0.87–1.33); p = 0.483	0.62 / 0.58	0.63 / 0.56	χ^2 = 0.50; p = 0.481

Radiological status: pelvic or para-aortic findings. Outcome: pathological LN metastasis in any region, except for "Both regions examined", in which the outcome is pathological pelvic or para-aortic metastasis. CT model: radiological pelvic and para-aortic status; combined model: CT model plus $\log_2$ CA-125. Corrected AUCs from 1000 bootstrap resamples. High-grade serous and stage III–IV analyses are exploratory; FIGO stage is assigned postoperatively and partly reflects nodal status. All 32 patients with stage I–II disease were pathologically LN-negative (specificity of the reported-node criterion 69% (95% CI 51–82); of the size criterion 97% (95% CI 84–99)). aOR, adjusted odds ratio; AUC, area under the curve; CI, confidence interval; OR, odds ratio.

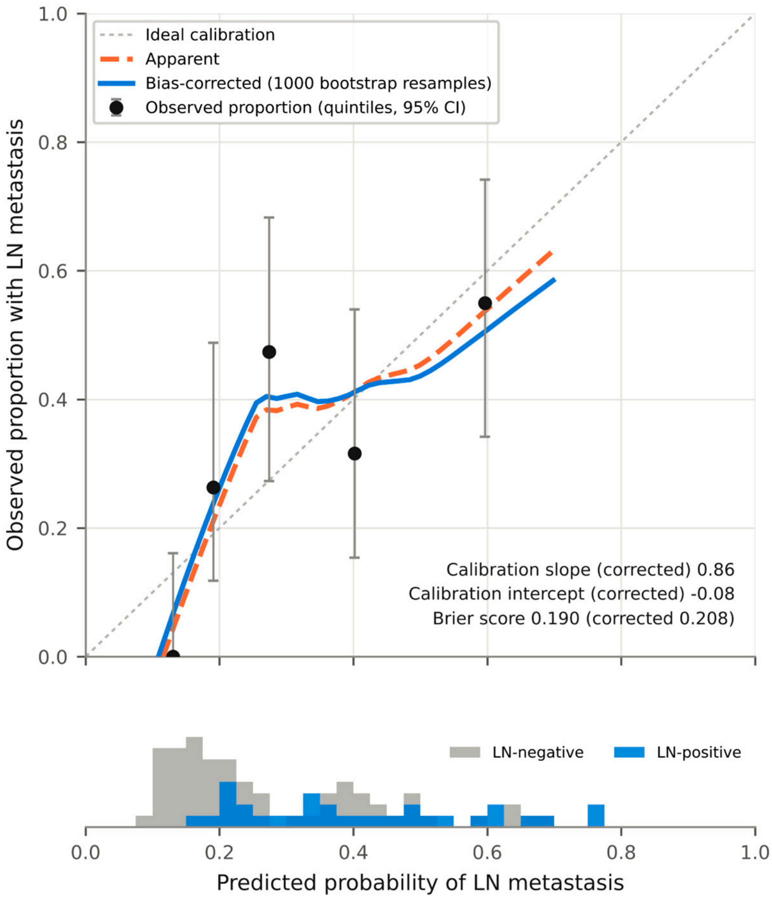

Figure S1. Calibration plot of the combined model (radiological pelvic and para-aortic status plus $\log_2$ CA-125). Apparent and bias-corrected (1000 bootstrap resamples) lowess calibration curves, observed proportions with 95% confidence intervals by quintile of predicted risk, and the distribution of predicted probabilities in pathologically LN-negative and LN-positive patients. Optimism-corrected calibration slope 0.86, calibration intercept −0.08; Brier score 0.190 (corrected 0.208).

Table S2. STARD 2015 checklist.

Section	Item	Checklist item	Reported in
Title or abstract	1	Identification as a study of diagnostic accuracy using at least one measure of accuracy	Title; Abstract
Abstract	2	Structured summary of study design, methods, results and conclusions	Abstract
Introduction	3	Scientific and clinical background, including the intended use and clinical role of the index test	Section 1; Section 4.4
	4	Study objectives and hypotheses	Section 1 (last paragraph)
Methods: study design	5	Whether data collection was planned before (prospective) or after (retrospective) the index test and reference standard were performed	Section 2.1 (retrospective)
Methods: participants	6	Eligibility criteria	Section 2.1
	7	On what basis potentially eligible participants were identified	Section 2.1
	8	Where and when potentially eligible participants were identified (setting, location and dates)	Section 2.1
	9	Whether participants formed a consecutive, random or convenience series	Section 2.1 (consecutive)

Section	Item	Checklist item	Reported in
Methods: test methods	10a	Index test, in sufficient detail to allow replication	Section 2.2
	10b	Reference standard, in sufficient detail to allow replication	Section 2.3
	11	Rationale for choosing the reference standard (if alternatives exist)	Section 2.3 (histopathology)
	12a	Definition of and rationale for test positivity cut-offs or result categories of the index test, distinguishing pre-specified from exploratory	Section 2.2 (reported node, primary; short axis >10 mm, secondary)
	12b	Definition of and rationale for test positivity cut-offs or result categories of the reference standard	Section 2.3
	13a	Whether clinical information and reference standard results were available to the performers or readers of the index test	Sections 2.1 and 2.2 (reports issued before surgery; extractor not blinded)
	13b	Whether clinical information and index test results were available to the assessors of the reference standard	Section 2.3 (routine care; blinding not documented)
Methods: analysis	14	Methods for estimating or comparing measures of diagnostic accuracy	Section 2.5
	15	How indeterminate index test or reference standard results were handled	Section 2.2 (no equivocal descriptions; one report without nodal statement coded negative)
	16	How missing data on the index test and reference standard were handled	Sections 2.1 and 2.2 (no missing measurements; no reference standard: excluded)
	17	Any analyses of variability in diagnostic accuracy, distinguishing pre-specified from exploratory	Sections 2.5, 3.3 and 3.5; Table S1
	18	Intended sample size and how it was determined	Section 2.5 (no a priori calculation; all eligible patients)
Results: participants	19	Flow of participants, using a diagram	Figure 1
	20	Baseline demographic and clinical characteristics of participants	Table 1
	21a	Distribution of severity of disease in those with the target condition	Table 2
	21b	Distribution of alternative diagnoses in those without the target condition	Table 2
	22	Time interval and any clinical interventions between index test and reference standard	Section 2.1 (≤2 months; NACT before CT in interval surgery)
Results: test results	23	Cross tabulation of the index test results (or their distribution) by the results of the reference standard	Figure 1; Tables 3 and 4
	24	Estimates of diagnostic accuracy and their precision (such as 95% confidence intervals)	Tables 4 and 5; Table S1
	25	Any adverse events from performing the index test or the reference standard	Not applicable (routine clinical care)
Discussion	26	Study limitations, including sources of potential bias, statistical uncertainty and generalisability	Sections 4.2 and 4.5
	27	Implications for practice, including the intended use and clinical role of the index test	Section 4.4; Section 5
Other information	28	Registration number and name of registry	Not registered (retrospective study)
	29	Where the full study protocol can be accessed	Not available
	30	Sources of funding and other support; role of funders	Funding statement (no external funding)

Checklist items from Bossuyt et al., BMJ 2015;351:h5527.
